# Strategy-making Process and Firm Performance in Iranian Pharmaceutical Industry

**Published:** 2019

**Authors:** Seyyed Abolfazl Abolfazli, Mehdi Mohammadzadeh, Farzad Peiravian, Jafar Zonoozi, Hesam Sharifnia, Ali Vasheghani

**Affiliations:** a *Department of Pharmacoeconomics and Pharmacy Management, School of Pharmacy, Shahid Beheshti University of Medical Sciences, Tehran, Iran.*; b *Department of Business Management, Faculty of Economic and Management, Urmia University, Urmia, Iran.*

**Keywords:** Strategy-making, Partial least square structural equation modeling, Smart PLS, Pharmaceutical companies, SPACE mode

## Abstract

This study investigated the relationship between main dimensions in the strategy-making process, environmental complexity, and performance in pharmaceutical companies of Iran. Results argue that pharmaceutical companies like other industries place differing emphasis on strategy-making and employ different modes of strategy-making. It offers a typology of different modes of strategy-making that most likely exist in pharmaceutical companies and hypothesizes how this typology relates to performance. It then describes the results of an empirical study of the strategy-making processes of pharmaceutical companies. The structural equation analysis of the data from 31 pharmaceutical companies indicates that there are simplistic, adaptive, entrepreneurial, participative, and SPACE (Strategy making Process According to Complexity Environment) modes of strategy-making in pharmaceutical companies; of these modes, SPACE mode exhibited a significant relationship with performance.

## Introduction

The main challenge for managers making strategy is encountering with an uncertain future. In studying strategy-making process, researches emphasize the critical role of sense making as a collective process for dealing with uncertainties about the business, market, and environment (1, 2). The strategic management process means defining the organization›s strategy. It is also defined as an applied field of business whose survival and growth does not only depend on its theoretical sophistication and the rigor of its methods*. *Although previous reviews and studies show that firms are specialized users of strategic management, they are more successful than ordinary users (firms that have not yet acquired strategy-making skills) (3, 4). It is recognized that one of the most important factors of ability of a firm to improve its performance is strategy-making process; on the other hand, the strategy making process that a firm uses may have to realize its strategic intent (5). Strategy-making has been studied in several areas in different sciences. Many studies have examined overall effects of formal planning systems (6), strategic decision processes (7), and strategy implementation processes in firm performance (8). Nevertheless, what often has been neglected is hypothesis testing related to organization-strategy-making processes (9). Strategy-making process has been proposed as a critical process that impacts overall firm performance, planning performance, entrepreneurial intensity, and planning flexibility (5, 10). Scholars have investigated the strategy-making processes of firms and their impact on firm performance for more than four decades (11). Research suggests the positive influence of strategy making processes on the firm performance.

Pharmaceutical industry is specified as the most profitable industry with Research and Development (R and D) activities (12). Few studies have investigated and developed models of strategy-making in pharmaceutical companies specifically where researchers have studied strategy-making in small firms; generally, the research tends to be prescriptive and focuses on discovering the degree to which formal strategy-making processes are employed in these firms (13). Pharmaceutical managers and other knowledge-based pharmaceutical companies should pay special attention to develop strategy-making and infrastructure to boost their performance (14)*. *The importance of strategy-making in pharmaceutical companies and the lack of research in the field of strategy-making in the pharmaceutical companies confirm the necessity of this research. The pharmaceutical industry faces a diverse stakeholder groups, including patients, health professionals, the media, regulators, political authorities, and the general public (15). In addition, the success or failure of strategy-making in pharmaceutical companies affects the health system policy at general level. Therefore, the present study aimed to identify strategy-making processes pharmaceutical companies use and also explain which approaches are more likely to succeed under different circumstances. To this extent, it provides a snap-shot of the state of strategy-making in pharmaceutical companies. It does not pretend to offer an all-inclusive coverage of the field, but rather an exploratory investigation into a field of study that has been under-investigation in pharmaceutical companies. Strategy-making process is defined as an organizational-level method of executing the phases of strategic management (*i.e.*, strategy formulation, environmental scanning, implementation, evaluation, and control).

The present study set to review divergent findings of previous models and empirical studies of the strategy-making process and examines relationships between the styles of strategy-making process and financial performances in pharmaceutical companies and tests related hypotheses.

This study sought to answer two basic questions:

Q1: Are pharmaceutical companies employing all or some of the simplistic, adaptive, entrepreneurial, participative, and SPACE strategy-making processes?

Q2: Which strategy making style has the most impact on performance?

Firstly, this paper reviews the theoretical basis of the subject; and then offers a concept pattern resulting from theoretical bases. Subsequently, research methodology and process of data analysis are presented, and then the results are presented. 

Strategic management is an interesting field of study that has evolved in creative and unpredictable ways almost half a century ago. Strategic management process is a method by which managers conceive of and implement a strategy that can lead to a sustainable competitive advantage (13). Strategy-making has been defined as a process that is a deliberate intervention to reconfirm or redesign, involving decision making by top managers and/or other organization members (14). Different firms make strategies in different ways including a set of modes to strategy-making process (15), presented as complementary and called a typology of strategy-making processes. Empirical studies of strategy-making process have conceptualized strategy-making as level of formality, planning horizon, or comprehensiveness (16). Research has shown consistent and inconsistent relationships with formal planners achieving higher performance (17, 18); these conflicting research results may be due to poor operationalization of planning construct, and its various components may confound results when tested individually (19). Following definitions by Dess and Beard, complexity is an environmental variable for strategy-making process typology. Complexity is defined as the heterogeneity and concentration of environmental elements, but the concept has been conceptualized as either the variety or range of an organization’s activities or geographic concentration-dispersion of firms within an industry (20). This article examines strategy-making process according to complexity environment (SPACE) mode with regard to other modes of strategy-making in pharmaceutical performance.

However, a model of strategy-making process must be parsimonious, yet makes use of multiple indicators which are grounded in theory and represent the most salient aspects of the process. For this discussion, strategy-making process is defined as an organization-level mode of operation utilized in the phases of strategic management: information processing and interpretation, strategic ends and means formulation, and strategy implementation. This section attempts to explore the existing typologies of strategy-making for modes relevant to pharmaceutical companies. At the end, a typology of strategy-making process for pharmaceutical companies is provided in the form of Hypothesis 1.

*Various strategies making typology*

Previous classification systems of strategy-making models have several scenario typologies such as multiple strategy-making process models (20) or multiple models, simultaneously; when it is required (21). The strategy-making process models are based on two main rational dimensions *vs.* emergent dimension which have been consistently separated (21-23). In addition, the previous classification system models are differentiated by various factors such as actors involved (21), resolving the conflicts between stakeholders (24), the importance of organizational coalitions power and politics (25), the creation of legitimacy and gaining commitment among organizational members (26), and a top-down *vs.* bottom-up approach (23). Eventually, considering the vital role of the environment in strategy-making process, all authors of previous classification systems suggested the choice of particular strategy-making process models based on the environment. The environment was described as the organizational context, the level of complexity and dynamism, the level of uncertainty, the existence of external influence, the rate of change, the strength of the competition, and time constraints (21). Since the present study is based on a contingency approach, it is assumed that the appropriate strategy-making process for a firm depends on its environment (25). Therefore, the contingency perspective is the basis for a division of the strategy-making process into several modes. Six modes of strategy-making typologies including rational, adaptive, participative, simplistic, command, and entrepreneurial are found. In the following, dimensions of strategy-making are described.

Rational mode: The rational mode of the strategy-making process shows that top*-*level managers determine broad strategies for the organization in general, and the detail of that strategy emerges over time through the actions of the employees of the firm. Some of the authors such as Hart (27) agree that the rational mode of strategy-making is very important to firms while this article suggests that the rational mode may not be relevant to firms at all. Conversely, other modes of strategy-making may be more appropriate to firms.

Adaptive mode: The strategy-making process in the mode is defined as fragmented strategies that develop over time to maintain flexibility (28). The environment of firms in this model is described as high environmental uncertainty, highly complex, and unpredictable. Firms in order to survive in the environment must be highly adaptive to changes in the external environment and to requirements of internal integration. This study argues that the adaptive mode of strategy-making could improve the quality and reduce the cost to enhance flexibility and maximize the use of accumulated knowledge, and also it argues how pharmaceutical companies should respond to regulatory changes.

Participative mode: participative mode depends on a high level of involvement in strategy-making and often through political processes; the symbolic mode relies on a strong organizational culture, defined by the vision, basic philosophy, and values of the firm (29). 

Simplistic mode: Simplistic strategy-making combines the command and symbolic modes of strategy making. On the other hand, Lumpkin and Dess describe the simplistic mode of strategy-making as characterized by ‘single-mindedness, narrowly construed decision-making, and excessive attention to a specific internal strength or external opportunity’.

Command mode: Hart (1992) describes the command mode as a mode of strategy making in which ‘a strong individual leader or a few top managers exercise total control over the firm’. In this mode, a strong individual leader or a small top team exercises total control over the firm (30). 

Entrepreneurial mode: The entrepreneurial mode identifies one approach in which employees can be involved in the strategy-making process, actually, the opposite of the command mode. In this mode, employees generate ideas, and therefore influence the strategic direction of the firm. This mode implies independent behavior by innovative employees who are encouraged and sponsored by top management to experiment and take risks (29). This mode indicates that strategy-making in firms occurs mainly from the bottom of the firm upwards or through teamwork, but in very small firms, it is unlikely to be the norm supposedly because of the strong influence of the firm owner (15, 16). This paper argues that it is likely that strategy may be generated emergently by innovative employees in some firms without strong direction from the owner or manager of the firm. Therefore, it is likely that the entrepreneurial mode exists in such enterprise.

*SPACE strategy making process*

Dynamism has been proposed as one factor to impact the strategy-making process in many theoretical models (26, 29). In contrast, the results from three decades of strategy research using dynamism as a construct are equivocal (9). There have been consistent problems in the operationalization of dynamism as well as substantial measurement error and other research design problems. While most of the studies in strategic planning have utilized self-report measures of volatility, objective measures of the environment could be incorporated into models to more effectively test direct and moderating effects. While dynamics do not have a strong efficiency alone, research argues that environmental complexity directly impacts the amount and nature of information required by decision-makers. In addition, most researchers have argued that complexity is expected to have a direct impact on organizational structure and processes (24).

In the pharmaceutical industry, due to continuous changes in the business environment related to domestic and foreign laws and regulations governing and intensive monitoring, complexity is created in the environment. This paper aims to confirm the existence of appropriate strategy-making in pharmaceutical company and to investigate its relationship with firm performance in a complicated environment*.*

In a separate study, the same researchers found seven dimensions of SPACE (Strategy making Process According to Complexity Environment) model. These dimensions were extracted from the study of strategy-making processes of a successful pharmacy company:

Continuous environmental change

Learning team 

Dialog

Improvising strategies

Strategy portfolio

Contemporary implementation of strategies

Agent activity

Given that none of these four modes (adaptive, entrepreneurial, participative, and simplistic) have been studied in a complex environment and harsh regulated environment, some of the results show that these methods have not sufficient effectiveness in terms of environmental complexity and uncertainty. So, a model which is appropriate to the situation called “SPACE” is introduced. In the following, the influence of each quad mode (namely the adaptive, entrepreneurial, participative, and simplistic modes) and fifth mode (SPACE) are analyzed.

Using Dess, Lumpkin, and Covin’s (1997) approach to hypothesize the strategy-making processes used by firms, a synthesis of the above research suggests that:

H1: Small firms will employ all or some of the simplistic, adaptive, entrepreneurial, SPACE, and participative strategy-making processes.

**Table 1:** Kolmogorov-Smirnovtestreturns

**Research variables**	**Z**	**Sig.**	**Normality**
Performance	1.766	0.004	Non normal
Strategy-making	1.465	0.027	Non normal
Simplistic	1.614	0.011	Non normal
Adaptive	1.447	0.030	Non normal
Entrepreneurial	1.484	0.024	Non normal
Participative	1.463	0.028	Non normal
SPACE	1.202	0.111	Normal

**Table 2:** Standardized weights results

**Latent variables**	**Question**	**Factor loadings**
	Q1	0.587
	Q2	0.720
	Q3	0.518
SPACE	Q4	0.590
	Q5	0.634
	Q6	0.712
	Q7	0.758
	Q8	0.909
Adaptive	Q9	0.562
	Q10	0.859
	Q11	0.764
Entrepreneurial	Q12	0.585
	Q13	0.656
	Q14	0.536
	Q15	0.648
	Q16	0.654
	Q17	0.585
Participative	Q18 Q19	0.676 0.760
	Q20	0.744
	Q21	0.662
	Q22	0.775
	Q23	0.775
	Q24	0.919
	Q25	0.590
	Q26	0.878
Simplistic	Q27	0.787
	Q28	0.819
	Q29	0.562
	Q30	0.848
	Q31	0.742
	Q32	0.688
Strategy-making	Q33	0.729
	Q34	0.762
	Q35	0.625
	Q36	0.720
	Q37	0.790
	Q38	0.615
	Q39	0.548
Q40		0.586
Performance Q41		0.862
Q42		0.953
Q43		0.925
Q44		0.870
Q45		0.623

**Table 3:** Cross loads results

**Question**		**SPACE**		**Adaptive**		**Entrepreneurial**		**Participative**		**Simplistic**		**Strategy-making**		**Performance**	
Q1		0.587		-0.023		-0.018		0.420		0.457		0.270		0.315	
Q2		0.720		0.130		0.137		0.521		0.519		0.340		0.433	
Q3		0.518		0.184		0.192		0.335		0.466		0.349		0.378	
Q4		0.590		-0.071		-0.058		0.266		0.244		0.175		0.110	
Q5		0.634		0.223		0.225		0.230		0.396		0.357		0.410	
Q6		0.712		-0.015		-0.009		0.435		0.513		0.249		0.322	
Q7		0.758		0.129		0.137		0.131		0.147		0.096		0.090	
Q8		0.319		0.908		0.902		0.342		0.117		0.424		0.316	
Q9		0.287		0.562		0.058		0.325		0.146		0.460		0.254	
Q10		0.308		0.859		0.581		0.603		0.073		0.382		0.297	
Q11		0.319		0.108		0.764		0.342		0.117		0.424		0.316	
Q12		0.290		0.070		0.585		0.314		0.166		0.454		0.260	
Q13		0.308		0.576		0.656		0.603		0.073		0.382		0.297	
Q14		0.395		0.353		0.333		0.536		0.228		0.395		0.380	
Q15		0.600		0.326		0.328		0.648		0.587		0.356		0.301	
Q16		0.253		0.416		0.422		0.654		0.104		0.292		0.202	
Q17		0.577		0.399		0.384		0.585		0.226		0.337		0.287	
Q18		0.506		0.259		0.269		0.676		0.381		0.517		0.260	
Q19		0.285		0.489		0.494		0.760		0.107		0.410		0.310	
Q20		0.471		0.179		0.189		0.744		0.604		0.209		0.197	
Q21		0.235		0.477		0.482		0.662		0.106		0.365		0.268	
Q22		0.223		-0.041		-0.029		0.775		0.487		0.068		0.023	
Q23		0.616		0.290		0.271		0.775		0.538		0.318		0.285	
Q24		0.493		0.508		0.496		0.360		0.919		0.375		0.261	
Q25		0.518		-0.077		-0.071		0.414		0.590		0.262		0.345	
Q26		0.638		0.323		0.307		0.693		0.878		0.406		0.280	
Q27		0.713		0.381		0.382		0.287		0.787		0.545		0.579	
Q28		0.755		0.243		0.256		0.377		0.819		0.508		0.621	
Q29		0.214		0.221		0.229		0.517		0.562		0.545		0.280	
Q30		0.591		-0.082		-0.071		0.395		0.848		0.297		0.293	
Q31		0.423		0.371		0.361		0.332		0.212		0.742		0.428	
Q32		0.565		0.263		0.275		0.426		0.422		0.688		0.537	
Q33		0.404		0.374		0.369		0.321		0.254		0.729		0.393	
Q34		0.496		0.618		0.605		0.346		0.223		0.762		0.536	
Q35		0.534		0.241		0.252		0.470		0.385		0.625		0.594	
Q36		0.404		0.212		0.219		00.399		0.587		0.548		0.720	
Q37		0.588		0.099		0.109		0.441		0.601		0.467		0.790	
Q38		0.268		0.488		0.475		0.267		0.515		0.467		0.615	
Q39		0.156		0.215		0.225		0.349		0.375		0.412		0.548	
Q40		0.341		0.320		0.325		0.385		0.563		0.591		0.586	
Q41		0.042		-0.299		0.057		0.064		0.026		-0.0141		0.862	
Q42		0.078		0.084		0.091		0.039		-0.282		-0.125		0.953	
Q43		0.039		-0.012		0.097		0.087		-0.022		-0.132		0.925	
Q44		0.277		0.056		0.230		0.248		0.197		-0.000		0.870	
Q45		0.062		0.064		0.005		0.034		0.014		-0.228		0.623	

**Table 4:** validity and reliability of the model

**Latent variables**	**AVE**	**CR**	**R** **2**	**Cronbach's alpha**	**GOF**
Strategy-making	0.508	0.837	-	0.756	0.531
Adaptive model	0.650	0.843	0.258	0.704	
Entrepreneurial model	0.645	0.840	0.305	0.800	
Participative model	0.784	0.886	0.334	0.859	
SPACE	0.945	0.825	0.489	0.761	
Simplistic model	0.497	0.829	0.194	0.730	
Performance	0.722	0.896	0.518	0.764	

**Table 5:** Correlation coefficients and divergent validity

**Latent variables**	**(1)**	**(2)**	**(3)**	**(4)**	**(5)**	**(6)**	**(7)**	$\sqrt[{2}]{\mathrm{AVE}}$
Strategy-making (1)	1							0.714
SPACE (2)	0.683	1						0.969
Adaptive model (3)	0.654	0.563	1					0.806
Simplistic model (4)	0.538	0.621	0.264	1				0.704
Performance (5)	0.581	0.641	0.673	0.434	1			0.849
Participative model (6)	0.606	0.597	0.647	0.667	0.397	1		0.885
Entrepreneurial model (7)	0.624	0.558	0.672	0.268	0.281	0.646	1	0.803

**Table 6:** The results of a hypothesis test

**The main hypothesis of research**	**β**	**T**	**R** **2**	**Sig.**	**Type of Relationship**	**Result**
Strategy-making → Performance	720/0	423/9	518/0	000/0	+	approved

**Table 7:** Investigate the relationship between modes and performance

**Independent variable**	**Dependent variable**	**β**	**T**	**R** **2**	**Result**	**Type of Relationship**
SPACE		0.699	8.577	0.489	approved	+
Adaptive		0.508	7.211	0.258	approved	+
Entrepreneurial	Performance	0.553	6.783	0.305	approved	+
Participative		0.578	7.613	0.334	approved	+
Simplistic		0.441	4.655	0.194	approved	+

**Figure 1:**
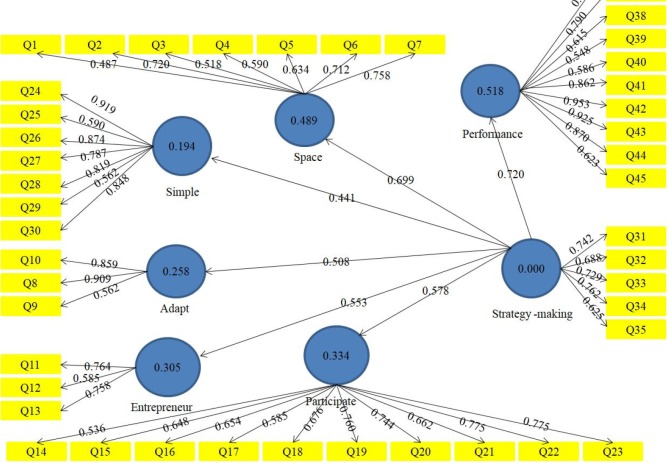
Strategy-making performance (standardized weights)

**Figure 2:**
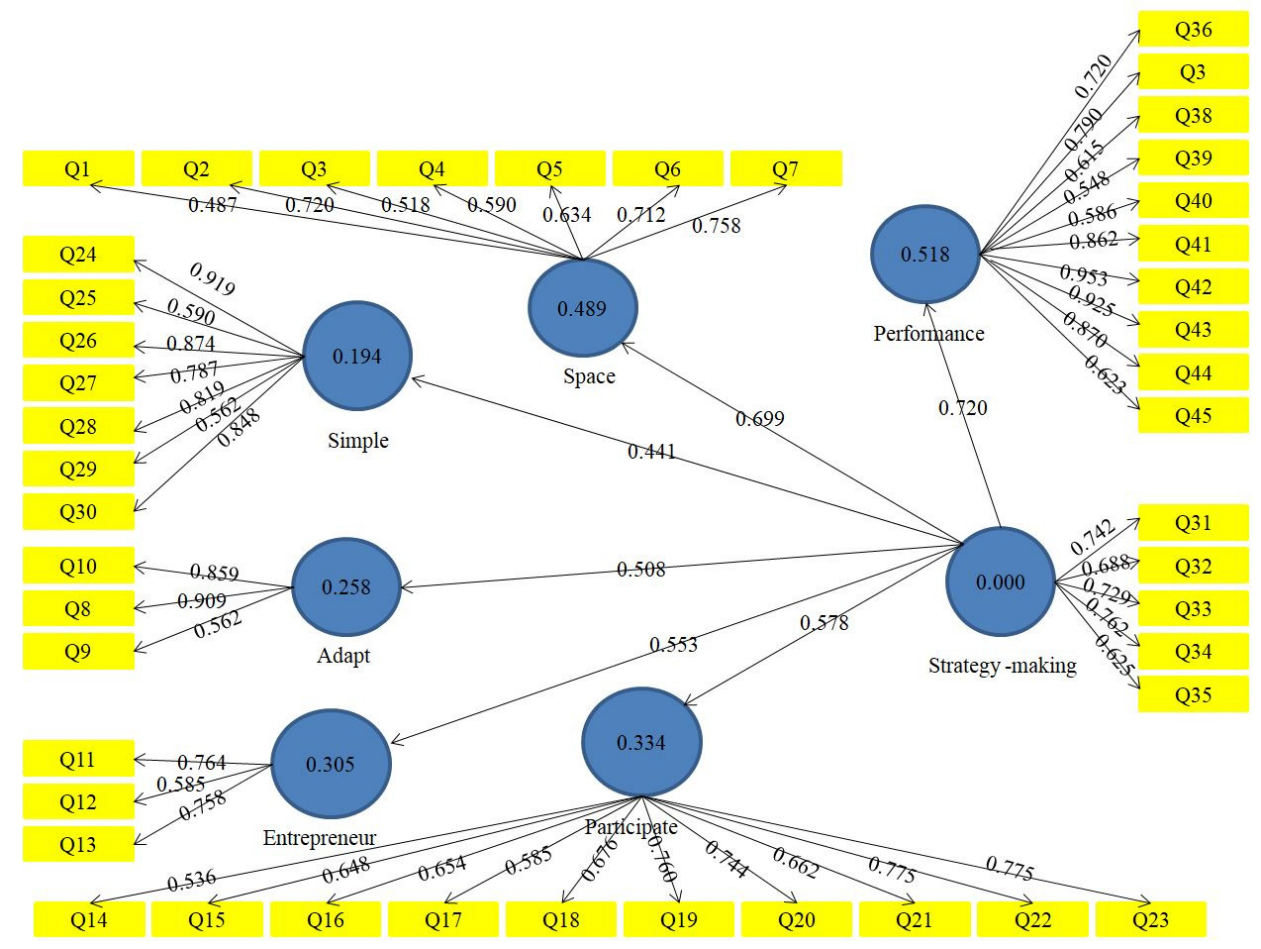
Strategy-making performance (|T-Value|)

*Strategy-making processes and firm performance*

Many studies have reported the effect of strategy-making processes on firm performance. Some researches indicated a positive relationship with formal planners achieving higher performance (31). For example, a study of 97 small firms in Canada showed that processes that are more rational in nature (more explicit strategies, longer planning horizons, and more detailed decision analysis) will be strongly associated with firm performance. Another study of Dutch small firms indicated that formal processes will influence performance, and performance will in turn lead to more formal strategy-making processes (11, 32). In general, it seems as if the support for a strong relationship between formal strategy-making and firm performance is quite conclusive. Other authors have studied the relationship between adaptive strategy-making and firm performance. Barney suggests that adaptive strategy-making is a rare and inimitable process that will lead to competitive advantage (33). Hart found in a study on 916 firms of all sizes and industry sectors that the adaptive mode of strategy-making is more highly associated with firm performance than the rational and generative (entrepreneurial) modes (28). But Van Gelderen *et al.* found that adaptive strategy-making does not only lead to poor performance. Participative strategy-making also receives some attention in this regard (32). Parnell and Crandall (2001) raised the possibility that participative decision-making techniques may improve decision quality and therefore organizational effectiveness (34). Frese, van Gelderen, and Ombach (2000) found that critical point (participative) strategy-making is highly related to firm success (35). 

The results of an empirical study of the strategy-making processes of SMEs in New Zealand indicated that participative approaches to strategy-making exist in these SMEs and may have a significant relationship with firm performance (36).

There has also been much debate about the performance outcomes of an entrepreneurial mode of strategy-making. Beaver and Jennings (2000) posited that the ‘relationship between enterprise performance, management actions (or inaction), and the value and contribution of strategy is extremely tenuous and very difficult, if not impossible, to demonstrate conclusively’. Entrepreneurial strategy-making will lead to growth and profitability for the firm or it may impede performance (37).

Now, this question arises: Which strategy making style leads to the best performance firms?

According to the contingency theory, the optimal strategy of a firm depends on many factors, such as availability of qualified employees and other resources (external factors), quality of the current employees, and the goals and strategic behavior of the business owner (both internal factors). Also sustainable competitive advantages are often referred to as important determinants for the selection of the strategy. These factors differ largely between firms. Hence, it is not possible to derive one most favorable strategy for a certain group of firms. Each company has to find its own optimal strategy, determined by external and internal factors of the firm. This theory states that firm performance is mainly determined by the quality of the strategy and the role of the entrepreneur in the formulation of strategy instead of the direction of the strategy. This paper defines SPACE strategy-making as a mode of strategy-making in which strategies are the result of the inclusion of various stakeholder views in different stages of strategy-making process. In some organizations, the interaction between especial internal stakeholders leads to political activity.

It can therefore be argued that:

H2: Firms that employ the SPACE modes of strategy-making will outperform those that employ other modes of strategy- making.

## Experimental

An empirical study was conducted to test the hypotheses set out earlier. A questionnaire was designed to elicit the five modes of strategy-making and firm performance. Although a variety of contingency variables were also included in the questionnaire, the present study focused only on the strategy-making and firm performance aspects. In this section, a brief overview of the survey instrument and data-analysis is provided.

*Data collection*

The questionnaire was developed based on the literature review and guides of the expertise on their field. Questionnaire containing scales was identified through a literature review mailed to 31 Phamacutical companies in Iran, selected companies listed on the stock exchange, and off-exchange. Pharmaceutical companies had activities in the field of drug manufacturing (chemical, herbal, and biological). A total of 45 usable questionnaires were returned, entered into an Excel datasheet, and analyzed by SPSS 11.5 and Smart PLS. 

*Measurement instrument*

Strategy-making mode was measured with the Hart scale as modified by Dess *et al.* (14, 28). This scale was originally developed by Hart to test strategy-making modes based on two dimensions: (1) top management ‘intentionality’, and (2) [organizational] actor “autonomy”. Dess *et al.* modified the scale and found four modes resulting from factor analysis, and the fifth mode (SPACE mode) was developed. Their scale consisted of 45 items, all measured based on the Likert scale from 1 (Strongly Disagree) to 5 (Strongly Agree). 

The dependent variable (firm performance) was measured using the financial performance. Respondents had to indicate the ‘importance’ of ten financial measures, namely sales level, sales growth rate, cash flow, return on shareholder equity, gross profit margin, net profit margin from operations, profit to sales ratio, return on investment, ability to fund business growth from profits, and overall firm performance on a 5-point Likert scale. Thereafter, they were asked to indicate their satisfaction with the firm’s performance for the same ten performance measures. The ‘satisfaction’ scores were multiplied by the ‘importance’ scores and aggregated in order to compute a weighted average performance index for each firm. The higher the aggregate score on this relative index, the better the perceived level of firm performance.

*Data-analysis*

The data were investigated to ensure that they satisfied the underlying assumptions for parametric testing. It was concluded that the assumptions for random sampling, normality, linearity, and homoscedasticity were satisfied. The measurement instrument was also tested for reliability and validity. Further data analyses were conducted using structural equation modeling. Measurement model for the five modes of strategy-making in Hypothesis 1 and 2 was developed and analyzed with partial least squares (PLS) (38). Five modes of strategy-making were the result of a process in which alternative models of modes of strategy-making were compared through SEM. 

## Results

The measurement model was developed and analyzed as a confirmatory factor analysis (CFA). PLS was used as an evolving approach to structural equation modeling (SEM). Kolmogorov-Smirnov test was used to assess the normality of the variables. In all tests, statistical hypothesis is as follows:

H0: normal data (data come from a normal population)

H1: data are not normal (data are not from a normal population)

*Kolmogorov-Smirnov test results*

The amount of significant level in all variables except the SPACE mode variable was lower than the amount of error 0.05 (Table 1). PLS estimation method determines the coefficients so that the resulting model has the most power of interpretation and explanation, *i.e.* the model with the highest accuracy could predict the final dependent variable.

The strategy-making performance (standardized weights) can be observed in Figures 1 and 2. Table 2 reports the standardized weights results for the measurement model.

In order to analyze the structure of the questionnaire and discover the constituent elements of each structure, factor loadings are used. The results of factor loadings variables are summarized in Table 2. All values of factor loads more than 0.5 were operating loads. So, the alignment of questionnaire for measuring concepts at this valid stage can be presented. In fact, the results indicated what the researcher intended to measure by the questioner was realized by this tool. The relationship between structures or hidden variables was invoked, an indication that has higher load factor and more importance than other indications. 

Table 3 shows the cross loads; the highest amount of loadings for each indicator is the structure of that index, and for the rest of the structures, it shows the lower factor loadings and any structure or latent variable which provided most of the load factor from its own parameters. Thus, latent variables’ models are distinguished enough.

*Computing convergent validity and reliability of the model*

Table 4 shows validity indexes and reliability for all variables of the study. Accordingly, AVE is equal to the average variance; CR is equal to the composite reliability; R^2 ^is equal to the coefficient of variation. Discriminant validity was also used, *i.e.* each structure is finally appropriate differentiation markers measured in terms relative to other structures provide models. Simply, each indicator only measures its structures and their combination in such a way that all structures are well separated. Assuming an average variance extracted, it is found that all observed structures with extracted average variance are higher than 0.5. Indicators’ composite reliability (CR) and Cronbach›s alpha were used, and is the requirement of approval higher reliability of these indicators from the 0.7 amount. These entire coefficients are higher than 0.7, showing the reliability of the measuring tools.

*Computing diverging credit*

Table 5 explores the correlation coefficients and divergent validity. The requirements for the approval of diverging validity are high content of second root of average variance from all correlation coefficients of related variable with the rest of variables. The positive coefficient indicates positive relation, and negative coefficient indicates negative and reverse relationship between two variables. All coefficients at the level of error lower than 0.05 were considered meaningful.

## Discussion

The questionnaire included two general variable processing strategies (including modes of 1. simplistic, 2. adaptive, 3. entrepreneurship, 4. participative, and 5. SPACE strategy) and performance variable which is edited, as well as the reliability and validity of variable were assessed. After validation and standardization of the questionnaire, based on statistical samples, the questionnaires were distributed among the population, and collected data were analyzed using the SPSS software. Collected data were used to test hypotheses on significant obstacles and differences among them. In Table 6, the main hypotheses and the test results in PLS software are provided in brief.

Based on the results of structural equation coefficients, t-value for this parameter is equal to 9.423 and (-1.96 < 9.423 > 1.96). Therefore it can be concluded that assumption is confirmed with 95% confidence and strategy-making impact on performance. Based on the positive path coefficient can be concluded that strategy-making significant positive impact on performance. Determination coefficient for performance is equal to 0.518. So strategy-making explains the 51.8% of performance changes

Confirmatory factor analysis found that the simplistic, adaptive, entrepreneurial, participative, and SPACE modes of strategy-making were important modes of strategy-making that Iran pharmaceutical companies exhibit. In particular, the SPACE mode identified by the data showed an idyllic mode of a firm in which a large amount of cooperation, teamwork, and values drive the strategy-making process. This indicates common modes such as simplistic, adaptive, entrepreneurial, and participative modes have considerable effect on performance. New modes, including SPACE, can have a significant impact on performance. Also, the cause of the incidence of SPACE strategy-making in Iran pharmaceutical companies is questioned. In fact, the possibility is raised that strategy-making practices may differ between countries and companies. Investigations on the possible relationship between all common modes of strategy-making and firm performance in previous studies suggest that sometimes all or some of the modes are likely to be associated with high performance. 

This study suggested that SPACE leads to improved decision-making, thus improved performance. This study not only suggests that SPACE strategy-making necessarily leads to success, but also it is possible that more successful firms are likely to delegate decision-making to the most appropriate levels of the firm.

Based on the results Table 7, the following suggestions are offered:

1: "Strategy making has positive and significant impact on performance", approved with hardness effect of 0.720. In principle, the work of strategy making is to understand and address the issue of competition. Often, managers take the competition easy like that it occurs only among direct competitors. But, competition for profitability is only one of the five factors that play a key role in the competition customers, suppliers, newcomers, and alternative products. Intense competition based on five factors is the approval of the industry structure and forms competitive nature of interactions within an industry. Therefore, it has been suggested that managers pay attention to the strategy making factor for business survival and check its dimensions and implement it properly in the company.

2: "SPACE mode has positive and significant impact on performance", approved with hardness effect of 0.699. This variable has the greatest impact among five dimensions. According to this hypothesis, the suggestion is that firms due to the complexity in the environmental SPACE mode to prioritize. Today›s complex environment requires a complex strategy.

3: "Participative strategy making has positive and significant impact on performance", approved with hardness effect of 0.578. The main purpose of the partnership is the use of others’ forces, transparency of issues, and revealing hidden aspects of the issues for making decision and to do better implementation of things. According to this hypothesis, it is suggested that in all matters decided through consultation, we can benefit from the partnership.

4: "Entrepreneurship strategy-making has a positive and significant impact on performance", approved with hardness effect of 0.553. Entrepreneurship is a perspective of change and gives opportunity and chance. Entrepreneurship strategy making is an exploitation and localization of opportunities, finding needed resources creating added value, social and financial networking, having scientific knowledge creating financial, social and artistic capital, having risk management, having assertion and determination in facing adversity, and containing creativity and innovation. It is recommended to flow entrepreneurship in all levels of organization to identify opportunities in all moments.

5: "Adaptive strategy-making has positive and significant impact on performance", approved with hardness effect of 0.508. Companies must learn how to do new things quickly and consistently change value offer in the form of products and services offered to customers. Of course, this feature should be beyond developing possibility of rapid change in products and organization services. So, that should work with agility and speed and change more important components of the business model, strategies, and processes. In the new models based on the ability to adapt to modern analysts, they recommend that managers lead their strategic analysis beyond organizational boundaries.

6: "Simplistic strategy making has positive and significant impact on performance", approved with hardness effect of 0.441. It is suggested that as we take steps in a complex environment with complex strategies, simple and practical strategies should be followed to gain the most benefit.

The measurement model describes the data well; and Hypothesis 1 can therefore be accepted. So, Iranian Pharmaceutical companies is likely to employ the simplistic, adaptive, entrepreneurial, participative, and SPACE modes of strategy-making. On the other hand, by accepting the second hypothesis, it is indicated that SPACE mode exhibits a significant relationship with performance.

There are many possible limitations when reading the results of the present investigation regarding strategy-making modes in the measurement model suggested, which are comprehensive but certainly not exhaustive. Results of the data analysis show that some strategy-making modes are more strongly related to performance. The data cannot be interpreted as indicating that firms not performing well are not engaged in strategy-making at all. As another limitation, the data were collected from pharmaceutical companies of Iran; further researches in other settings or countries are recommended to confirm the results.

## Conclusion

It is concluded that SPACE strategy-making is an important mode of strategy-making used by pharmaceutical companies. When examining this mode more closely, it was found that SPACE strategy-making includes the following features: attention to new opportunities, considering competitors, and adapting continuous changes; on the other hand, employees share the company›s prospects and follow it as an ideology. Because of the variety of pharmaceutical products and the high number of competitors in the pharmaceutical industry, SPACE strategy-making impacts positively pharmaceutical performance. Hence, SPACE strategy-making is significantly related to firm performance, suggesting that considering complexity in the strategy-making process is a suitable approach for pharmaceutical companies to ensure that the decisions resulting from the process will improve the competitive position of the firm. The present study suggests, therefore, that pharmaceutical companies’ owners*/*managers who are concerned with the development of strategy-making processes in their firms can expect high benefit. Overall, the results indicated that strategy processing has a positive impact on performance. This effect was observed in all aspects of strategy. Overall strategy processing was at a high level. Improving the level of strategy enhanced the performance. Of all aspects of the strategy processing examined, the complex strategy aspect allocated the greatest impact to itself. Decisions and integration activities were performed to develop effective strategies and to control the results. Strategy processing confirms the need for collective mind and mass reflection, and this emphasizes participation as an important factor in strategy processing. Other aspects with different and positive effects on performance are brought respectively.
